# The Probiotic *Escherichia coli* Strain Nissle 1917 Combats Lambdoid Bacteriophages *stx* and λ

**DOI:** 10.3389/fmicb.2018.00929

**Published:** 2018-05-29

**Authors:** Susanne Bury, Manonmani Soundararajan, Richa Bharti, Rudolf von Bünau, Konrad U. Förstner, Tobias A. Oelschlaeger

**Affiliations:** ^1^Institute for Molecular Infection Biology, University of Würzburg, Würzburg, Germany; ^2^Ardeypharm GmbH, Herdecke, Germany

**Keywords:** probiotic, *E. coli* Nissle 1917, EHEC, Shiga toxin producing *E. coli*, *stx*-phages, lambda-phages, lambdoid prophage, LamB

## Abstract

Shiga toxin (Stx) producing *E. coli* (STEC) such as Enterohemorrhagic *E. coli* (EHEC) are the major cause of foodborne illness in humans. *In vitro* studies showed the probiotic *Escherichia coli* strain Nissle 1917 (EcN) to efficiently inhibit the production of Stx. Life threatening EHEC strains as for example the serotype O104:H4, responsible for the great outbreak in 2011 in Germany, evolutionary developed from certain *E. coli* strains which got infected by *stx2*-encoding lambdoid phages turning the *E. coli* into lysogenic and subsequently Stx producing strains. Since antibiotics induce *stx* genes and Stx production, EHEC infected persons are not recommended to be treated with antibiotics. Therefore, EcN might be an alternative medication. However, because even commensal *E. coli* strains might be converted into Stx-producers after becoming host to a *stx* encoding prophage, we tested EcN for *stx*-phage genome integration. Our experiments revealed the resistance of EcN toward not only *stx*-phages but also against lambda-phages. This resistance was not based on the lack of or by mutated phage receptors. Rather it involved the expression of a phage repressor (*pr*) gene of a defective prophage in EcN which was able to partially protect *E. coli* K-12 strain MG1655 against *stx* and lambda phage infection. Furthermore, we observed EcN to inactivate phages and thereby to protect *E. coli* K-12 strains against infection by *stx*- as well as lambda-phages. Inactivation of lambda-phages was due to binding of lambda-phages to LamB of EcN whereas inactivation of *stx*-phages was caused by a thermostable protein of EcN. These properties together with its ability to inhibit Stx production make EcN a good candidate for the prevention of illness caused by EHEC and probably for the treatment of already infected people.

## Introduction

Foodborne illness caused by enteropathogenic bacteria are a great concern on population health. Among those the hemolytic uremic syndrome and bloody diarrhea are symptoms caused by foodborne pathogenic *Escherichia coli* (*E. coli*) strains, the so-called STEC strains (Conway, 2015). The first report of a STEC infection goes back to the year 1982 when the serotype O157:H7 was responsible for two outbreaks of hemorrhagic colitis in the USA (Mead and Griffin, 1998; Pennington, 2010). The main source of the STEC hazard is a toxic protein which is closely related to the Stx from the gram-negative bacterium *Shigella dysenteriae* (Fraser et al., 2004). Two Stx types are known to be produced by STEC strains of which Stx2 is reported to lead to a more severe disease progression than Stx1 (Eklund et al., 2002). Research on the mode of action of Stx revealed that the two component AB5 toxin binds to the globotriaosylceramide (Gb3) receptor of eukaryotic cells and blocks protein synthesis due to ribosome inactivation by the subunit A, which results in cell death by apoptosis (Tesh, 2012; Melton-Celsa, 2014).

Since the first outbreak in 1982, STEC infections have been reported worldwide (Karch et al., 1999; Vally et al., 2012; Byrne et al., 2015), whereby also non-O157 STEC play an increasingly important role (Bettelheim, 2007). Most alarmingly, in the year 2011 a non-O157 STEC O104:H4 caused an epidemic in Germany which led to the death of 53 people (Bielaszewska et al., 2011; RKI, 2011). The appearance of non-O157 STEC strains can be traced back to the fact that Stx is encoded on a prophage (O'brien et al., 1984). Stx and *stx*-phages are both released from lysogenic *E. coli* upon the increase of RecA (Neely and Friedman, 1998; Fuchs et al., 1999). The released phages can infect other *E. coli* strains and thereby turn harmless commensal strains into dangerous lysogens (Schmidt et al., 1999; Schmidt, 2001). As antibiotic treatment induces the SOS response and thereby the increase of RecA in *E. coli* it is not the ideal treatment strategy as it could worsen the progression of disease (Zhang et al., 2000; Pacheco and Sperandio, 2012). In this context, it is of great interest to find alternative strategies to treat STEC infected patients and their close surroundings.

Probiotics are defined by World Health Organization as “live microorganisms which when administered in adequate amounts confer a health benefit on the host” (WHO, 2001). They are usually part of the healthy human gut flora and have been used to treat gastrointestinal disorders (Islam, 2016). A lot of research on gut microbiota strongly argues that antibiotics lead to long term alterations of a healthy gut (Jandhyala et al., 2015). In contrast, a healthy gut microbiota contributes to the protection against gut infections by pathogens. This could be related to the fact that some of the commensal bacteria can produce antimicrobial agents to suppress the growth of other microorganisms or can colonize the gut better than the pathogenic bacteria (Oelschlaeger, 2010; O'shea et al., 2012; Deriu et al., 2013). Thus, probiotics are earning attention as alternatives or supplements to antibiotics.

Bacteriophages are one of the most abundant members of the gut microbiota and their roles in maintaining the microbial diversity are still unclear (Łusiak-Szelachowska et al., 2017). However, phage resistance is an important safety aspect for probiotic strains to maintain their genetic stability in the gut (Mattila-Sandholm et al., 2002). In contrast, prophages are masked in the genome of bacteria and process to phages by bacterial lysis in case of stress conditions for their host (Mills et al., 2013). Phages are reported to carry key virulence factors (Brussow et al., 2004) and can therefore be considered as an important source for acquisition of pathogenesis in bacteria (Herold et al., 2005; Darmon and Leach, 2014). Hence, it is of high interest for the probiotic strains to be not susceptible for the conversion into a pathogenic strain by phages carrying virulence genes.

EcN is one of the most extensively studied non-pathogenic, commensal *E. coli* strain which forms the active component of the probiotic drug “Mutaflor.” It was isolated by Prof. Nissle in 1917 during the First World War from the feces of a soldier who did not develop infectious diarrhea, which was a widespread disease in the field (Nissle, 1918; Sonnenborn, 2016). EcN was shown to exhibit antagonistic activity against different pathogenic enterobacteria, both *in vivo* and *in vitro* (Nissle, 1925; Sonnenborn and Schulze, 2009). For over one century now, EcN has been successfully used in the treatment of various gastrointestinal diseases (Montrose and Floch, 2005; Schultz, 2008).

As an approach to find alternative treatment strategies for STEC infections, a lot of research has been focusing on effects of EcN on those pathogenic strains. It was shown that EcN interferes with the adherence of STEC strains to gut epithelial cells (Rund et al., 2013) and negatively influences the Stx production during *in vitro* culture studies (Reissbrodt et al., 2009; Mohsin et al., 2015). Moreover, pre-colonization of mice with EcN hindered the STEC strain EDL933 from gut colonization (Leatham et al., 2009; Maltby et al., 2013). However, application of EcN to prevent or treat EHEC infections can only be considered if this strain cannot be infected by *stx*-phages and cannot be turned into a Stx producing lysogen itself.

The objectives of this study were to (i) investigate the genome stability of EcN toward lambdoid phage infections, (ii) study the phage resistance, (iii) detect possible *stx*- and lambda-phage antagonistic effects of EcN and (iv) analyze the protective capacity of EcN.

## Materials and methods

### Bacterial strains

*E. coli* strains used in this study are listed in Table 1. All strains were grown in Luria-Bertani broth (LB) medium (10 g/l tryptone, 5 g/l yeast, 5 g/l NaCl) at 37°C.

**Table 1:** *E. coli* strains used in this study.

***E. coli* strain**	**Serotype**	**Description**	**Source**
*E. coli* Nissle 1917 (EcN)	O6:K5:H1	Non-pathogenic probiotic strain	Ardeypharm, Herdecke
SK22D	O6:K5:H1	Microcin deletion mutant of EcN	Patzer et al., 2003
SE15	O150:H5	Commensal strain	Japan Collection of Microorganisms (JCM)
IAI1	O8: K-	Commensal strain	Erick Denamur, University of Paris-Diderot
MG1655	OR:H48:K-	K-12 laboratory strain	Strain collection, IMIB Wuerzburg
MG1655R	OR:H48:K-	MG1655 harboring the pUC19 plasmid	Strain collection, IMIB Wuerzburg
MG1655R*pr*	OR:H48:K-	MG1655 harboring the pUC19 plasmid with the *phage repressor* (*pr*)	Strain collection, IMIB Wuerzburg
HB101	OR:H48:K-	K-12 laboratory strain	Strain collection, IMIB Wuerzburg
DH5α	OR:H48:K-	K-12 laboratory strain	Strain collection, IMIB Wuerzburg
EHEC EDL933	O157:H7	*stx1, stx2* harboring STEC strain	Ulrich Dobrindt, Münster
EHEC 1530/99 (HUSEC018)	O26:H11	*stx2* harboring STEC strain	Strain collection, IMIB Wuerzburg
EAHEC TY3730	O104:H4	*stx2* harboring STEC strain, Clinical isolate from a gastroenteritis patient (2011)	Holger Rohde, Hamburg
EAHEC TY3456	O104:H4	*stx2* harboring STEC strain, Clinical isolate from a HUS patient (2011)	Holger Rohde, Hamburg
EHEC 4392/97 (HUSEC033)	O145:H25	*stx2* harboring STEC strain	Ulrich Dobrindt, Münster
*E. coli* K-12 993 W lysogen	not determined	K-12 strain harboring lambda lysogen	Klaus Hantke, Tübingen

### Extraction of phages

The induction of the phage production was achieved by adding 1 μg/ml of mitomycin C (MMC/OMNILAB GmbH & Co. KG, Munich, Germany) to a lysogenic *E. coli* culture in its mid-log growing phase (OD_600_ 0.3-0.5). The culture was further incubated in the dark for 6 h/24 h at 37°C in a rotary shaker (200 rpm) before the phages were isolated by sterile filtration (0.22 μm PALL filter/VWR, Darmstadt, Germany) of the supernatant (9,400 × g, 5 min, RT). *stx*-phages for the mono-/co-/and tri-culture studies with MG1655 were extracted from a 24 h shaking STEC culture without the addition of MMC.

### Growth conditions

The OD_600_ of bacterial overnight cultures (ONC) was determined and the cells were collected by centrifugation at 9,400 × g for 5 min at RT. The bacterial pellet was resuspended in LB medium to obtain ~10^8^ CFUs/ml of the STEC strains and ~10^9^ CFUs/ml of the commensal and *E. coli* K-12 strains. 100 μl of the STEC strains or 100 μl of a phage extract were used to set up mono-/co-/or tri-cultures with 100 μl of EcN and/or K-12 strains in a 24 well plate. The cell numbers used for co- and tri-cultures of STEC/phages: commensals/SK22D: K-12 strain was 1:10:10 = 10^7^ CFUs or pfus/ml: 10^8^ CFUs/ml: 10^8^ CFUs/ml. Whenever the K-12 strains were used in co- and tri-cultures with *stx-*phages, 100 μl of a non-MMC induced phage lysate was used (~10^6^ pfus/ml). The MOI of *stx-*phages: commensals/SK22D: K-12 strain was 1:100:100 = 10^6^ pfus/ml: 10^8^ CFUs/ml: 10^8^ CFUs/ml. Each well was adjusted to a final volume of 1 ml with LB medium. The plates were incubated in a static manner for the desired amount of time at 37°C. The CFUs were determined by plating serial dilutions in 0.9% saline on LB agar plates with a selective antibiotic or on ECC plates (medco Diagnostika GmbH, Munich, Germany). The samples were sterile filtered (0.22 μm PALL filter/VWR, Darmstadt, Germany) for further analysis.

### Pathogenicity evaluation

The Stx production of the STEC strains was identified in the sterile filtrated samples with the Ridascreen® Verotoxin ELISA (r-biopharm, Darmstadt, Germany) at the time point of interest. To determine the phage titer, phage plaque assay (PPA) plates were prepared (Islam et al., 2012). Therefore, 200 μl of a MG1655 ONC (OD_600_ 2.0-3.0) were mixed with 1.5 μg/ml MMC, 20 mM CaCl_2_ and adjusted to 3 ml with 0.7% LB agar. The mixture was poured on top of a 1.5% LB agar plate. After hardening of the LB agar the number of phage particles in the filtrate was determined by plating serial dilutions in 0.9% saline on the MG1655 indicator plate.

### Killing of *E. coli*

To study the phage inactivation mechanism *E. coli* strains were killed with heat or 1% formaldehyde (FA). 24 h static cultures were pelleted at 13,000 × g for 5 min at RT and washed twice with 500 μl of 0.9% saline. The pellets were resuspended in either LB medium to obtain 10^10^ CFUs/ml and thereon heat killed for 1 h at 100°C or incubated in 1 ml 0.9% saline, 1% FA overnight at 4°C. The 1% FA fixed *E. coli* were washed twice with 500 μl of 0.9% saline (13,000 × g, 5 min, RT) and resuspended in LB medium to reach 10^10^ CFUs/ml. For a more precise active component determination, heat killed bacteria were digested with 1 mg/ml Proteinase K (Qiagen, Hilden, Germany) for 1 h at 37°C. The Proteinase K inactivated by heat at 100°C for 8 min. 100 μl of the differentially killed *E. coli* were mixed with 100 μl of phages, adjusted to 1 ml with LB medium and incubated for 24 h at 37°C in a 24 well system.

### Lysogeny PCR

For the detection of lysogenic *E. coli* strains bacterial colonies were pooled from LB-Agar plates and the cells were washed twice with 1 ml 0.9% saline (13,000 × g, 5 min, RT). In each wash step, the pellets were vortexed vigorously to get rid of the phages that were attached outside the cells. The cells were then diluted 1:100 in 0.9% saline. 2 μl of a heat boiled bacterial suspension or sterile filtrates (10 min, 100°C) were used as template in a 25 μl PCR reaction using the PCR Master Mix (Thermo Scientific, Darmstadt, Germany). *stx*-phages: *stx2* (Primer: stx2_fwd/stx2_rev), lambda-phages: (Primer: lambda_Q_fwd/ lambda_Q_rev) (Table 2).

**Table 2:** Oligonucleotides used in this study.

**Name**	**Sequence (5'–3')**	**Concentration [nM]**	**Amplicon size [bp]**	**Application**	**References**
1L2	AATGAACCAGATCCGTGTGA	500	103	Amplification of an EcN specific chromosomal region	Troge et al., 2012
1R2	CAGGTCCAAACGTAACAGTGC	500			
4L2	GGGCGATCGGAT TTAATCAT	500	186	Amplification of an EcN specific chromosomal region	
4R2	CGAGGACTCGGAGCTTACTG	500			
5L1	GCCTCTCGCAACTTAACGAC	500	232	Amplification of an EcN specific chromosomal region	
5R1	AGTTATCCAGCGTTGCCATC	500			
K-12_fwd	TTCCCACGGACATGAAGACTACA	500	969	Amplification of a K-12 specific genome region	Kuhnert et al., 1995
K-12_rev	CGCGATGGAAGATGCTCTGTA	500			
stx2_fwd	CTGGGTTTTTCTTCGGTATCCT	500	518	Amplification of *stx2A*	This study
stx2_rev	ACAGTGACAAAACGCAGAAC	500			
lambda_Q_fwd	GGAGAAGGCGCATGAGACTC	500	639	Amplification of Q late gene regulator of lambda phage	
lambda_Q_rev	GCTGCTAACGTGTGACCGCAT	500			
hcaT_fwd	ACAAACGCAGGCCAGAAAG	200	127	Real-Time PCR reference gene for *E. coli*	Zhou K. et al., 2011
hcaT_rev	GCTGCTCGGCTTTCTCATC	100			
1294_fwd	TCCGATTAGCAGGGCTTT	150	59	Real-Time PCR for the *phage repressor* gene	This study
1294_rev	CCGGGCGTTTTTTATTGGT	150			
lamb_fwd	ATGTCTGCTCAGGCAATGC	150	136	Real-Time PCR for the lambda phage receptor gene	This study
lamb_rev	CACATTCGTTGCCAAGACGG	200			This study
PR_P1_rev	ATTCGAGCTCGGTACCCGGTCCTGTGG ATTGATCCAGTA	500	410	Cloning the phage repressor gene (*pr*) into MG1655	This study
PR_P2_fwd	CAGGTCGACTCTAGAGGATCCACGCAT ACCTTTCAACTA	500			
M13_pUC19_fwd	GCAAGGCGATTAAGTTGGGT	500	593 (pUC19_*pr*), 224 (pUC19)	Screening the clones in pUC19	This study
M13_pUC19_fwd	CACCCCAGGCTTTACACTTT	500			

### Lysogeny detection

*E. coli* strains were tested for a possible integration of the lambdoid phages. Therefore, *E. coli* strains were incubated static at 37°C with and without lambdoid phages until the plaque forming units (pfus) reached zero (44 h for *stx*-phages and 120 h for lambda-phages). At these timepoints, we could not detect any pfu in the sterile filtrate of EcN coincubation by PPA. 1 ml bacteria were washed twice with LB medium (13,000 × g, 5 min, RT) and resuspended in 1 ml LB medium. The washed bacteria were incubated for 14–16 h with 1 μg/ml MMC to induce lysis. The supernatant of the bacteria was sterile filtered (0.22 μm PALL filter/VWR, Darmstadt, Germany) and incubated for another 24 h, 37°C, static with MG1655 to amplify the phage signal by MG1655 lysis. The final phage titer was detected with the phage plaque assay.

### RNA isolation

Total EcN bacterial RNA was isolated from 3 ml cultures after 3 or 16 h of a static incubation period with or without phages. The cultures were transferred to 6 ml of RNAprotect® Bacteria Reagent (Qiagen, Hilden, Germany) and the RNA was isolated with the RNase Mini Kit (Qiagen, Hilden, Germany) according to the manufacturer's instructions. To remove remaining DNA the isolated RNA was digested with DNase (2 U/ml New England Biolabs, Frankfurt, Germany) as recommended by the company. The RNA was purified by an ethanol precipitation. Therefore, the digested RNA was adjusted to a final volume of 180 μl with RNase free dH_2_O. 18 μl of 3 M sodium acetate and 1.33 μl of 15 mg/ml GlycoBlue (ThermoFisher, Darmstadt, Germany) were added followed by a gentle vortexing step. The sample was adjusted to 800 μl with ice cold 100% EtOH. After an overnight incubation at −20°C the RNA was collected during a 30 min, 13,000 × g at 4°C centrifugation time. The pellet was washed twice with 250 μl 70% ice cold EtOH (13,000 × g, 5 min, 4°C) and dissolved in 25 μl of RNase free dH_2_O. The RNA content was determined by measuring the absorbance at 260 nm at the NanoDrop 2000c spectrophotometer.

### RNA library preparation

Extracted RNA was depleted of ribosomal RNA using the Ribo-Zero rRNA Removal Kit for bacteria (Illumina) according to the manual. Depleted RNA was fragmented for 3 min at 94°C using the NEBNext Magnesium RNA Fragmentation Module. The RNA ends were repaired with two consecutive T4 PNK incubations (−/+ ATP) and an RppH treatment. Library preparation was performed according to the NEBNext Multiplex Small RNA Library Preparation Guide for Illumina. All adapters and primers were diluted 1:4 and 15 and 16 cycles of PCR were used, respectively. No size selection was performed at the end of the protocol. 12 libraries were pooled and sequenced on a NextSeq 500 with a read length of 75 nt.

### Analysis of deep-sequencing data

The quality of raw reads (Phred scores, number of duplicates and adapter) were assessed using FastQC (version-0.11.31; Andrews, 2010^1^). In order to assure a high sequence quality, the Illumina reads in FASTQ format were trimmed with a cut-off phred score of 20 by cutadapt (version-1.15) (Martin, 2011) that also was used to remove the sequencing adapter sequences. The following steps were performed using the subcommand “create,” “align,” and “coverage” of the tool READemption (Forstner et al., 2014; version 0.4.3) with default parameters. Reads with a length below 20 nt where removed and the remaining reads were mapped to the reference genome sequences (NZ_CP007799.2) using segemehl (Hoffmann et al., 2009). Coverage plots in wiggle format representing the number of aligned reads per nucleotide were generated based on the aligned reads and visualized in the Integrated Genome Browser (Freese et al., 2016). Each graph was normalized to the total number of reads that could be aligned from the respective library. To restore the original data range and prevent rounding of small error to zero by genome browsers, each graph was then multiplied by the minimum number of mapped reads calculated over all libraries. The differentially expressed genes were identified using DESeq2 version 1.16.1 (Love et al., 2014). In all cases, only genes with maximum Benjamini-Hochberg corrected *p*-value (padj) of 0.05, were classified as significantly differentially expressed. The data were represented as MA plots using R.

The RNA-Seq data presented in this work has been deposited at the NCBI Gene Expression Omnibus (Edgar et al., 2002) and can be accessed through GEO series accession number GSE109932 (https://www.ncbi.nlm.nih.gov/geo/query/acc.cgi?acc=GSE109932).

### Real-time PCR

Primers were designed to confirm the transcriptome results. The isolated RNA was diluted to 40 ng/μl with RNase free dH_2_O. 1 μl of the RNA served as template in the Power SYBR™ Green RNA-to-CT™ 1-Step Kit (ThermoFisher, Darmstadt, Germany). The thermal cycler conditions were: reverse transcription at 48°C for 30 min, activation of the polymerase for 10 min at 95°C, 40 cycles of 95°C denaturation for 15 s and 60°C annealing/elongation for 1 min followed by 15 s of 95°C denaturation, 15 s at 60°C elongation and 15 s at 95°C denaturation. *hcaT* was used as *E. coli* reference gene (Zhou K. et al., 2011). The fold change was calculated by the formula, foldchange = 2^(−ΔΔCq).^ Where, ΔΔCq = (Cq reference − Cq gene of interest)_control_ − (Cq reference − Cq gene of interest)_treated._ The quantification cycle (Cq) is defined as the timepoint at which the fluorescence of a sample first increases above baseline fluorescence.

### Cloning the phage repressor (*pr*) in MG1655

The *pr* was amplified from the EcN genome using the primers PR_P2_fwd/PR_P1_rev (Table 2). The amplicons were cloned into pUC19 using the In-fusion cloning Kit (Takara Bio Europe, France). The *E. coli* K-12 strain MG1655 was transformed with pUC19 and the pUC19 harboring the phage repressor (pUC19_*pr*). The transformants were screened with pUC19_M13 fwd/rev primers (Table 2) and the importance of this gene in the protection of MG1655 phage infection was determined with the phage plaque assay.

### Phage plaque assay for *E. coli* K-12 clones

Phage plaque assay plates were prepared with 200 μl of ONC of *E. coli* (OD_600_ 2.0–3.0) with appropriate antibiotics and 3 ml 0.7% LB agar (+/− 1 mM IPTG). The plates with 1 mM IPTG were incubated at room temperature for the induction of gene expression. After 2 h, the phage lysate was serially diluted and 10 μl drops were spotted on the plates and incubated at 37°C for 24 h.

### Transwell assay

To identify if direct cell to cell contact is necessary for EcN to reduce the Stx and *stx*-phage production of EDL933 the *E. coli* strains were separated by a transwell membrane (0.4 μm ThinCert^TM^ Cell Culture Inserts, Greiner Bio-One GmbH, Frickenhausen, Germany) in a 6 well plate. 250 μml of the *E. coli* culture that were processed as described in section Killing of *E. coli*, were transferred to the well or the insert and both compartments were filled to a final volume of 2.5 ml with LB medium. The analysis of the samples was performed after a 24 h static incubation time at 37°C.

### Statistical analysis

The experiments were performed independently for at least 3 times in duplicates. Data are shown as mean ± SD. For the statistical analysis of the experimental data we used GraphPad Prism® version 6.01. The *t*-test was used for the significance.

## Results

### EcN is resistant to lambdoid-phage infection

**Figure 1:**
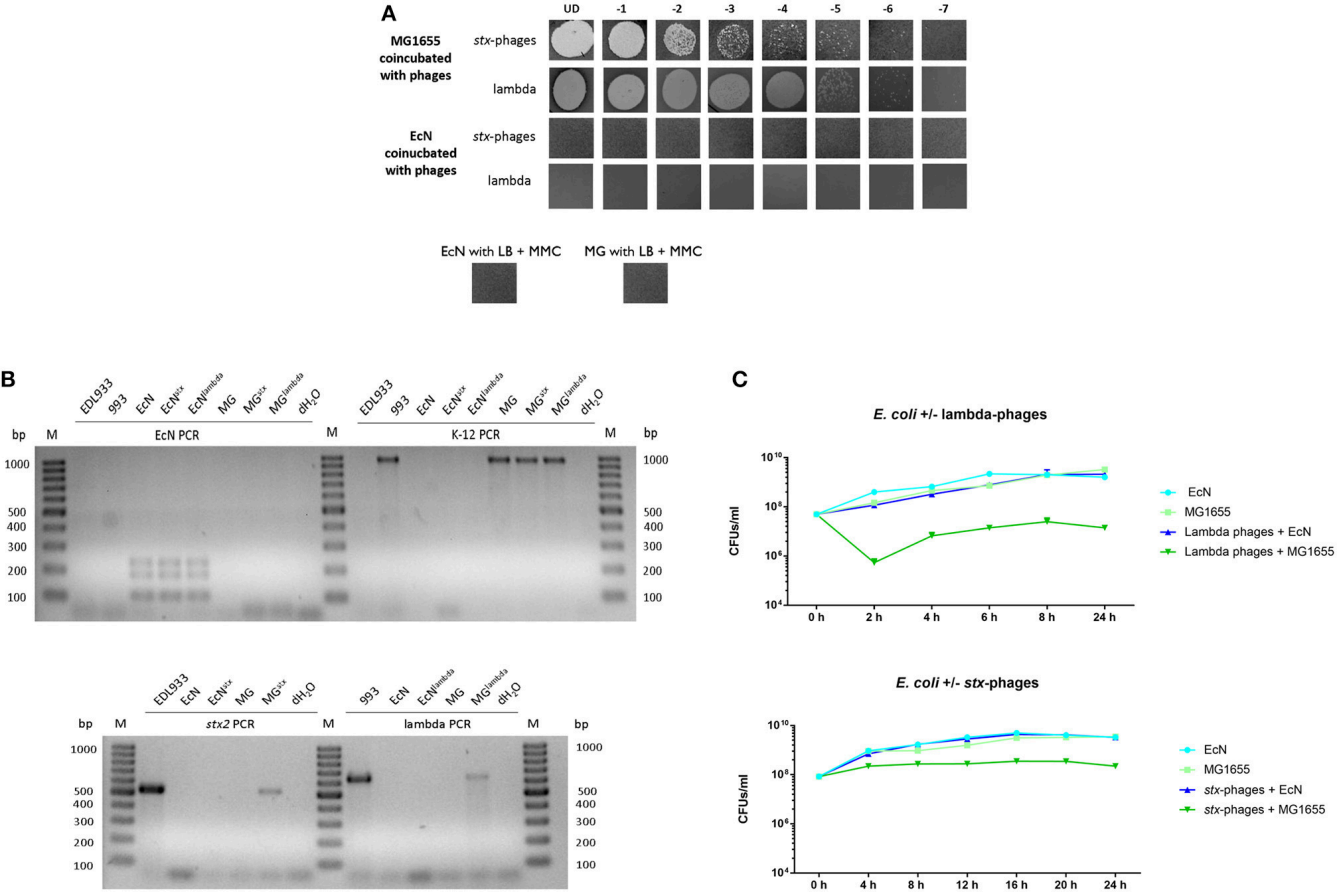
EcN test results for lysogeny by *stx*-/lambda -phages. **(A)** EcN and MG1655 were tested for lysogeny with *stx*-/lambda-phages. The *E. coli* strains were incubated with the respective phages for a period of 44 h (*stx*-phages, MOI: 100:1 = *E. coli*: phages) and 72 h (lambda-phages, MOI: 10:1 = *E. coli*: phages). Then they were forced to lyse by the addition of 1 μg/ml Mitomycin C (MMC). The phage signal was intensified by another incubation period with MG1655. The final phage titer was determined by a MG1655 Phage Plaque Assay (PPA). EcN and MG1655 + MMC served as negative control for lysogeny. **(B)** After a 24 h incubation period with (EcN/MG1655^*stx*/*lambda*^) or without (EcN/MG1655) *stx*- or lambda-phages EcN and MG1655 bacterial colonies were collected and tested for lysogeny with the stx2 primers or the lambda Q primers respectively. The strain identity was confirmed with the genome specific EcN primers 1L/R, 4L/R, 5L/R, and the K-12 primers. **(C)** The growth kinetics of EcN/MG1655 were determined in presence and absence of lambdoid phages during a period of 24 h. M, 100 bp ladder.

It is of clinical importance that EcN is not getting infected by *stx*-phages to be able to use it as a probiotic drug treatment during an EHEC infection or as a preventive medication. Lambdoid phages undergo lytic and lysogenic cycles inside the host cells during the infection (Casjens and Hendrix, 2015). Hence, we performed experiments to test if EcN is lysed/lysogenized upon phage-contact. To identify if EcN undergoes lysogenic infection, it was incubated with *stx*-phages from the STEC strain EDL933 and lambda-phages from the K-12 strain 993 as described in section Lysogeny Detection to allow the phage integration into bacterial genome (bacteria: phages = 10:1). In the next step, the cells were washed and induced with mitomycin C for phage production. It was clearly observed that in the sterile filtered supernatant of EcN no plaques could be detected even after mitomycin C induction (Figure 1A). Accordingly, there were also no lysogens detected by a phage specific PCR in EcN cells that had been co-incubated with lambdoid phages (Figure 1B). In addition, when the isolated phages were spotted on an EcN lawn there was no lysis observed, indicating that EcN did not undergo lytic infection (**Figure 3**). These results were also supported by the fact that the CFUs of EcN were not altered in the presence of phages compared to the EcN culture in the absence of phages (Figure 1C). In every experiment, the K-12 strain MG1655 showed successful lysis and lysogeny with lambdoid phages. Thus, our results demonstrated that EcN is resistant to lambda- and *stx*-phage infections.

### Contribution of prophage genes in EcN's resistance to lambdoid phages

**Figure 2:**
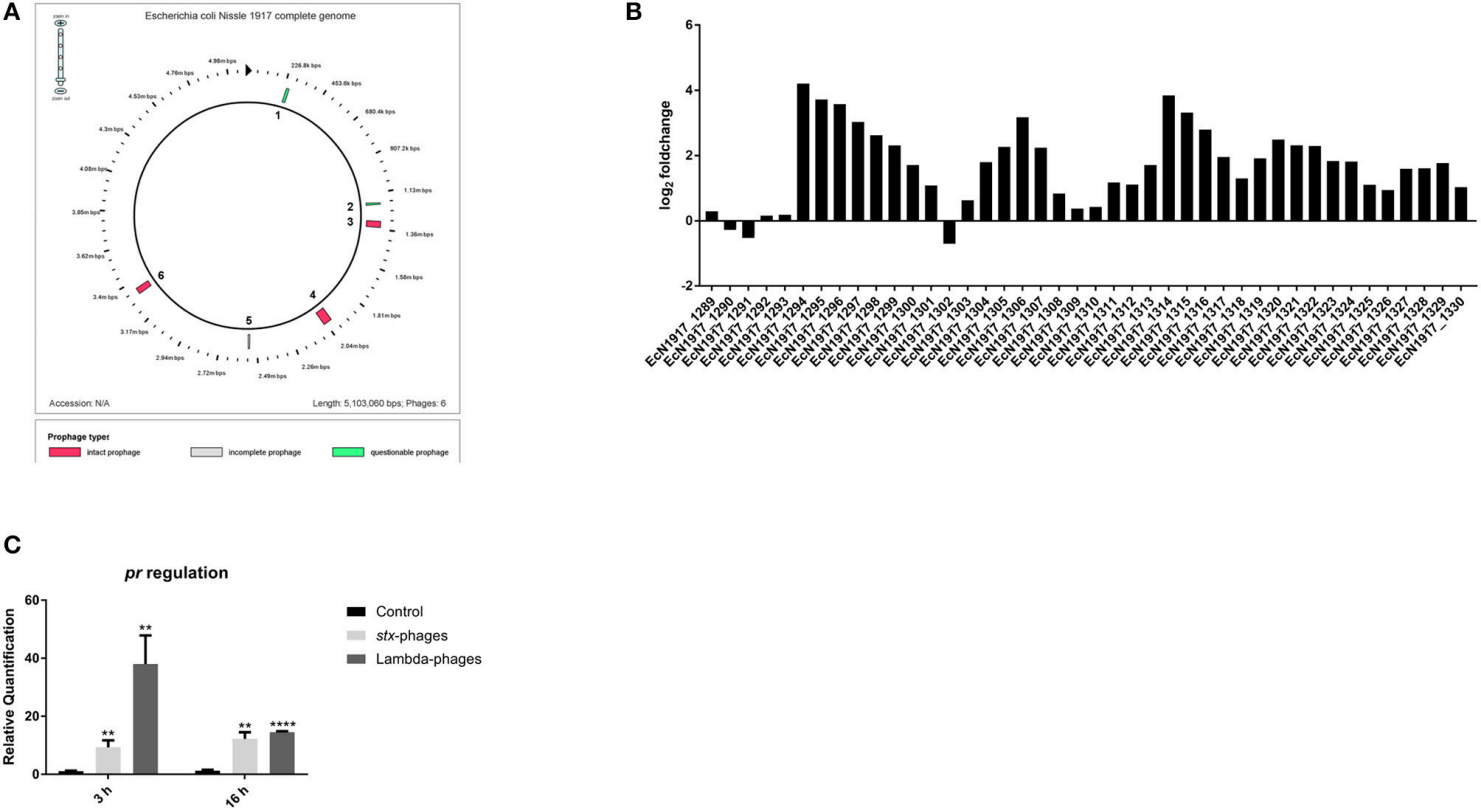
Phage protection analysis of EcN. **(A)** Prophages predicted in the genome of EcN by PHAST. **(B)** Transcriptomic results of the upregulated genes of a lambdoid prophage in EcN after 16 h of incubation with *stx*-phages. **(C)** Verification of the transcriptomic results of the highly upregulated gene EcN1917_1294: phage repressor (*pr*) via qRT-PCR with the primer pair after 3 or 16 h of incubation with both lambdoid phages types. *hcaT* was used as reference gene. Control = EcN + LB-medium, ^**^p < 0.01 and ^****^p < 0.0001.

**Figure 3:**
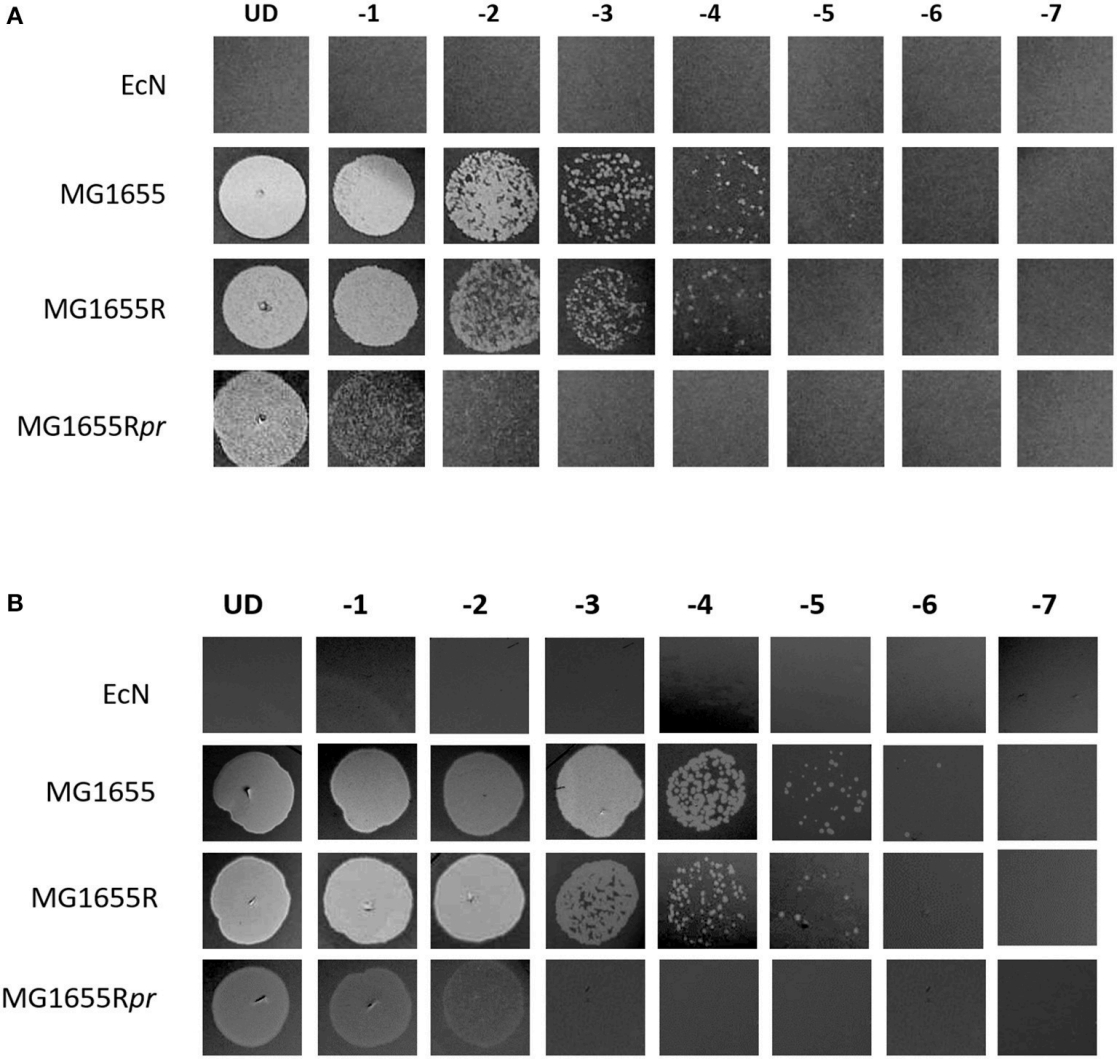
Determination of the protection in MG1655 recombinant strains toward lambdoid phage infection. MG1655 was transformed with pUC19 (MG1655R) with or without the phage repressor (*pr*) gene sequence of EcN (MG1655R*pr*). Serial dilutions of *stx*-phages **(A)** or lambda-phages **(B)** in 0.9% saline were dropped on EcN, MG1655 or MG1655 recombinants lawns. Lysis of the respective bacterial strain is visible as lysis zone or individual plaques. UD, undiluted, −1 to −7, 1:10 dilution series.

To elucidate the mechanism of EcN's resistance to lambdoid phage infections, the transcriptome of EcN was analyzed in the presence and absence of *stx-*phages (bacteria: phages = 10:1). The analysis revealed an upregulation of genes belonging to a prophage in EcN (Figure 2B). The prophage prediction in the genome of EcN was performed by PHAST—a PHAge Search Tool (Zhou Y. et al., 2011) and revealed that the upregulated prophage genes as being part of an intact lambdoid prophage (prophage 3) (Figure 2A, Table S1), whereas the genes of the other prophages depicted no to little regulation in to the presence of *stx*-phages (Figure S1). However, the predicted prophage 3 had a size of only 39.8 kb, whereas typically a lambdoid prophage would have a size of ~48 kb (Table S1) (Casjens and Hendrix, 2015). Also, prophages in EcN were neither induced by antibiotic nor by heat induction (data not shown). Hence, it could be speculated that this predicted prophage could be an inactive one. Based on the literature search about phage resistance (Ladero et al., 1998), we were interested in the highly upregulated gene, EcN_1294 coding for a phage repressor (Pr). A strong upregulation of this gene in EcN, incubated with lambdoid phages for 3 or 16 h, was further validated by qRT-PCR (Figure 2C). Contribution of the phage repressor in EcN's resistance to lambdoid phage infection was investigated by cloning it into the phage sensitive MG1655 strain. Results (Figure 3) indicated that isolated lambda- and *stx-* phages could plate on the recombinant MG1655 strain with the *pr* (MG1655R*pr*) 215- and 243-fold less efficiently than on the recombinant MG1655 strain with the pUC19 plasmid (MG1655R; vector control) (Figure 3). These results clearly indicate that *pr*, which belongs to a prophage 3 (Table S1) of EcN, plays a significant role in EcN's resistance to lambdoid phages.

### Effects of EcN on lambdoid phages

**Figure 4:**
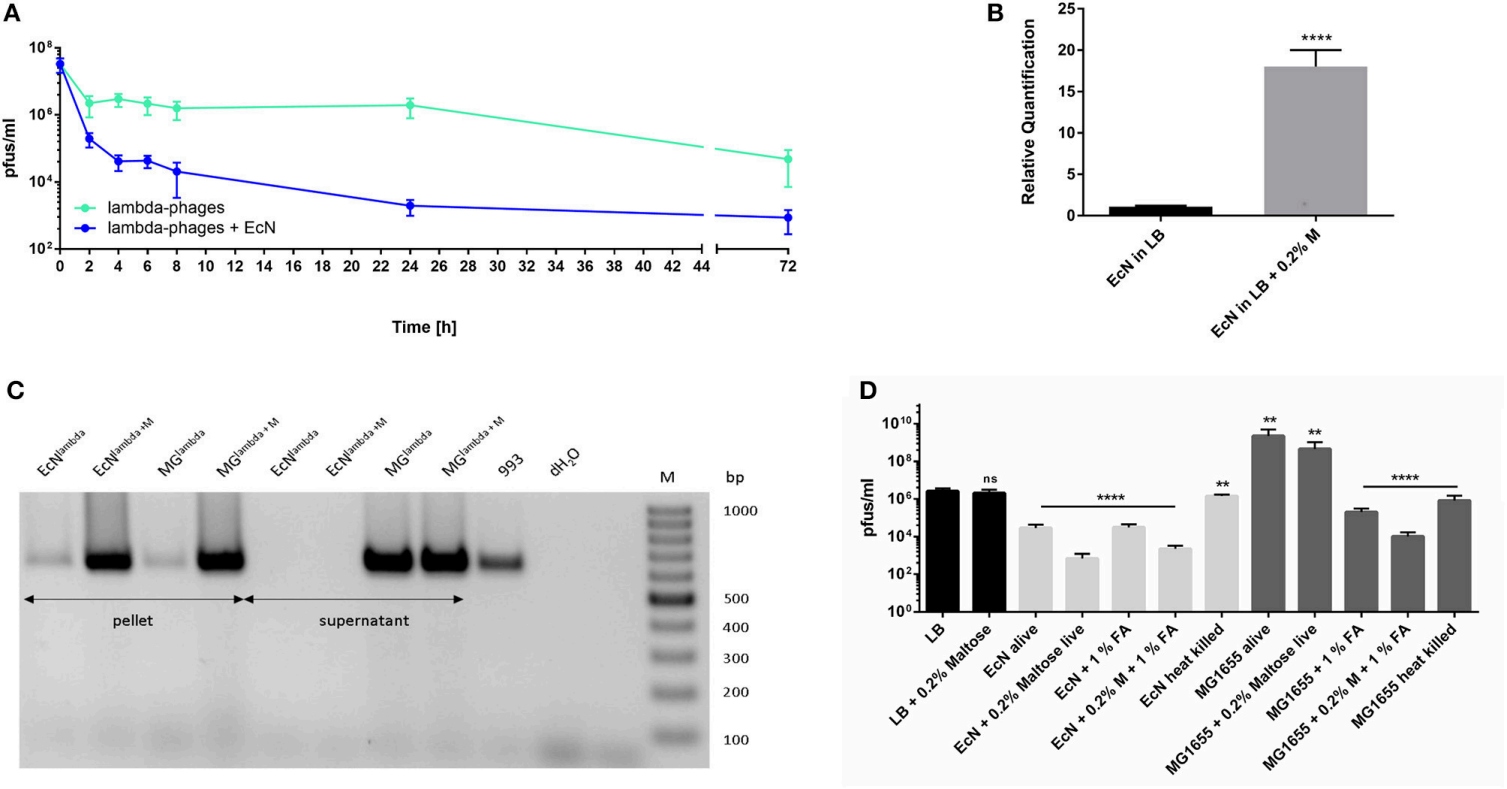
Phage inactivation studies of EcN and lambda-phages. **(A)** The lambda-phage pfus/ml kinetics were analyzed in the presence and absence of EcN in a period of 72 h. Phage titers were determined with the Phage Plaque Assays (PPA). **(B)** The *lamB* regulation of EcN was evaluated after 6 h incubation in LB medium with or without the addition of 0.2% maltose with qRT-PCR. *hcaT* was used as control gene. **(C)** To determine the phage localization, a PCR with the lambda Q primers was performed on the washed bacterial pellet or the sterile filtered supernatant of EcN or MG1655 after being incubated with lambda phages for 24 h in the absence or presence (+ M) of 0.2% Maltose. 993 is the *E. coli* K-12 strain that harbors lambda lysogen and it was used as positive control for this PCR **(D)** Heat killed or 1% formaldehyde (1% FA) fixed EcN or MG1655 were compared in their phage inactivation capabilities to the corresponding alive *E. coli*. Bacteria were incubated with or without 0.2% Maltose for 24 h, static before being killed. The lambda-phages were incubated for 24 h with the living or dead *E. coli* strains and subsequently the phage titer was determined with the PPA. ns, not significant, ^**^p < 0.01 and ^****^p < 0.0001.

Phage reduction was observed in the sterile filtered supernatants of EcN co-incubated with lambda- or *stx*-phages at a starting MOI of 1:10 (phages: bacteria). The kinetics were determined for both lambdoid phage types in the period from 0 to 72 h (Figure 4A, 5A). The lambda-phage titer was 550-fold reduced by EcN after 72 h of incubation and even 100,000-fold after 44 h in case of the *stx*-phages. The kinetics of the phage reduction by EcN varied significantly among the two lambdoid phage types. Therefore, we were interested in elucidating the distinct phage reduction mechanisms.

**Figure 5:**
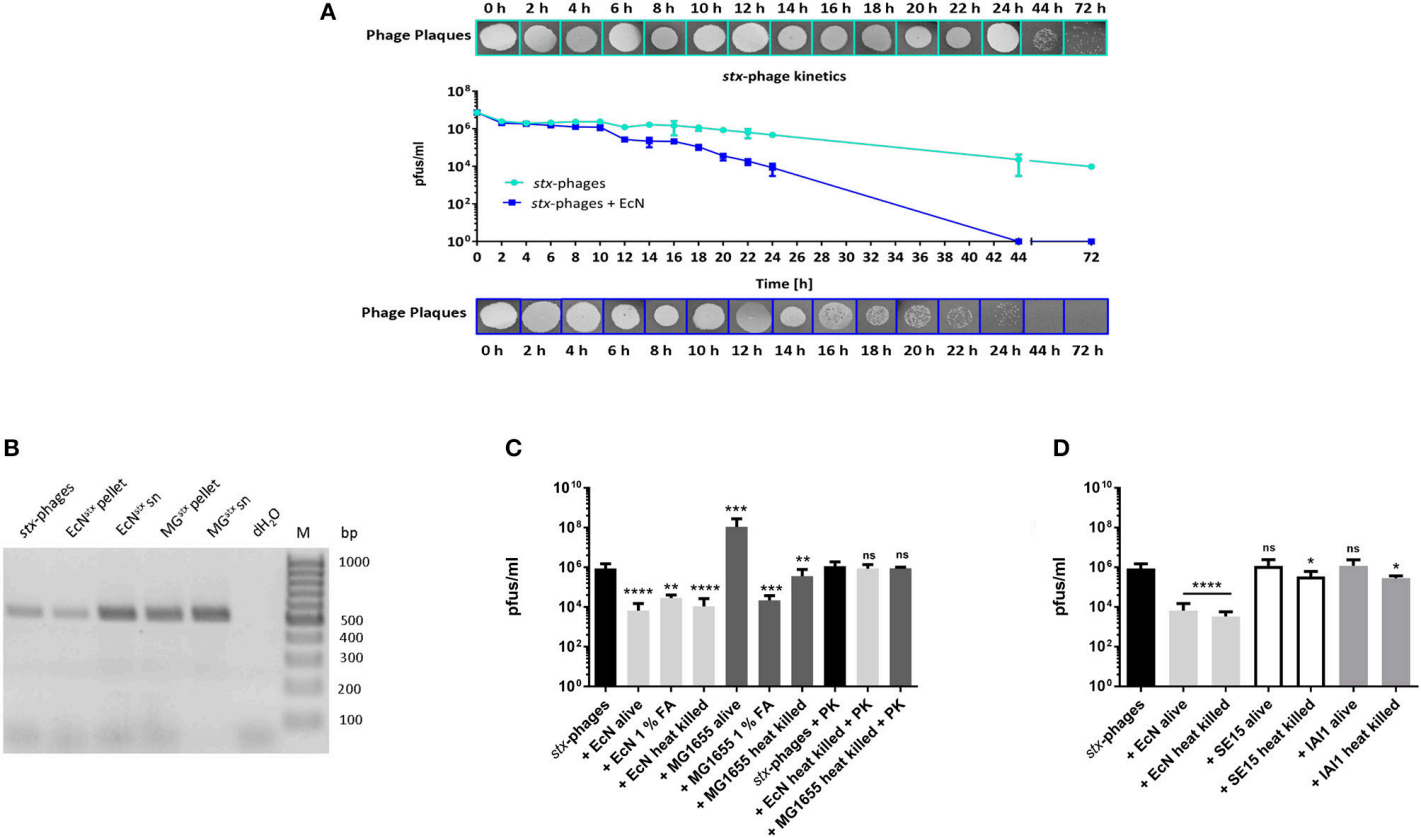
Phage inactivation studies of EcN and *stx*-phages. **(A)** The *stx*-phage pfus/ml kinetics were analyzed in the presence and absence of EcN in a period of 72 h. Phage titers were determined with the Phage Plaque Assays (PPA) **(B)** The phage localization PCR with the *stx2* primers was performed on the washed bacterial pellet or the sterile filtered supernatant (sn) of EcN or MG1655 after being incubated with *stx*-phages (EcN^*stx*^, MG^*stx*^)for 24 h. **(C)** Heat killed, heat killed and proteinase K (PK) treated or 1% formaldehyde (1% FA) fixed EcN or MG1655 were prepared as described in section Killing of *E. coli* and their phage inactivation capabilities were compared to the corresponding living *E. coli*. Bacteria were incubated for 24 h, static before being killed. The heat killed bacteria or the heat killed plus proteinase K treated bacteria were incubated for 24 h with the *stx*-phages and subsequently the phage titer was determined with the PPA. **(D)** Heat killed or living SK22D or the commensal control strains SE15 or IAI1 were incubated with the isolated *stx*-phages for 24 h before the pfus/ml were determined with a PPA. ns, not significant, ^*^p < 0.05, ^**^p < 0.01, ^***^p < 0.001, and ^****^p < 0.0001.

Lambda-phages are known to use LamB maltoporin as receptor (Randall-Hazelbauer and Schwartz, 1973; Chatterjee and Rothenberg, 2012). For this reason, we induced the LamB production by supplementing the medium with 0.2% maltose (Schwartz, 1976; Hoyland-Kroghsbo et al., 2013), which was verified by qRT-PCR analysis of the *lamB* regulation (Figure 4B). PCR analysis of the specific lambda-phage gene *Q*, in the pellet and the supernatant of EcN which were co-incubated with lambda-phages, depicted the phage DNA in the pellet, with heavily increased intensity in maltose supplemented medium (Figure 4C). As expected the phage inactivation by EcN after a 24 h incubation increased also from 100-fold to about 1,000-fold in the presence of maltose (Figure 4D). Further experiments with heat killed EcN resulted in a loss of the phage reduction capacity, whereas 1% formaldehyde fixed EcN reduced the lambda-phage titer to the same extend as the living bacterium. In addition, when maltose induced EcN were formaldehyde fixed the phage reduction was more pronounced. The results for the K-12 control strain MG1655 resembled EcN regarding the killed bacteria, however, it led to a 100–1,000-fold increase in the phage titer in case of co-incubation with alive MG1655 (Figure 4D).

In contrast to the lambda-phages, for which a phage reduction was depicted as early as 2 h after co-incubation, the *stx*-phage inactivation started only after 12 h of co-incubation with EcN. The *stx2* PCR located the phages rather in the supernatant than in the pellet of the EcN culture (Figure 5B). In the subsequent *stx*-phage reduction approach it was shown that even heat killed EcN were still able to lower the *stx*-phage titer by around 100 times like the living EcN (Figure 5C). This effect was lost in case of a Proteinase K digest of the heat killed EcN pellet. On the other hand, the phage susceptible control strain MG1655 increased the phage titer 1,000-fold, reduced the phage titer when being formaldehyde fixed and had no effect on the phage level when being heat killed. Two commensal strains SE15 and IAI1 were additionally investigated for having similar *stx*-phage inactivating effects as EcN alive or heat killed (Figure 5D). Both commensal strains lacked the phage inactivation which was observed for EcN alive and showed only a weak influence of 2.4-fold decrease by heat killed SE15 and a 3.5-fold decrease by IAI1 on the *stx*-phage infectivity when being heat killed.

Obviously EcN exhibited two different mechanisms for inactivating lambda- and *stx*-phages. Whereby, the latter seems to be an EcN specific attribute.

### EcN protects K-12 strains from phage infection

**Figure 6:**
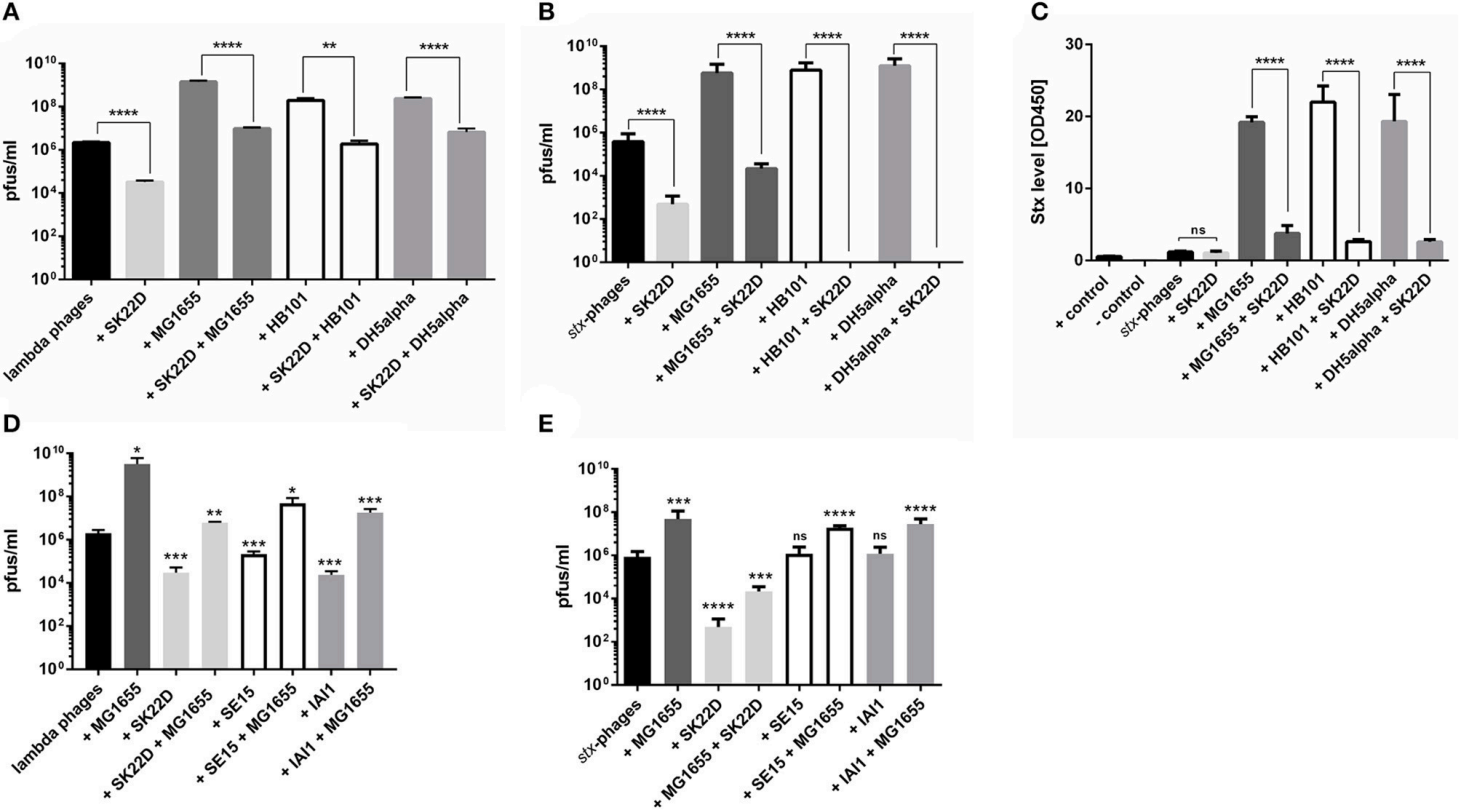
Protection of K-12 strain lambdoid phage infection by SK22D. Lambda-phages **(A)** or *stx*-phages **(B)** were incubated in mono-/co-/or tricultures with the microcin negative EcN strain SK22D and/or the K-12 strains MG1655, HB101 or DH5α at a starting MOI of 1:10:10 for 24 h, static. Afterwards, the pfus/ml were determined in the sterile filtrate with a Phage Plaque Assay (PPA). 24 h pfus/ml for HB101/ DH5α + SK22D + *stx*-phages: 0 pfus/ml **(C)** In case of the *stx*-phage experiment the Stx level of the filtrate was determined with the Ridascreen® Verotoxin Elisa. Lambda-phages **(D)** or *stx*-phages **(E)** were incubated in mono-/co-/or tricultures with SK22D, the commensal control strains SE15, IAI1 and MG1655 at a starting MOI of 1:10:10 for 24 h, static before the pfus/ml were determined with the PPA. LB Medium was used as negative control. ns, not significant, ^*^p < 0.05, ^**^p < 0.01, ^***^p < 0.001, and ^****^p < 0.0001.

To test the probiotic capabilities of EcN, the microcin negative EcN mutant SK22D was used to exclude the bacterial killing effects of the antimicrobial peptides (Patzer et al., 2003). lambda- or *stx*-phages were incubated at a starting MOI of 100:100:1 (K-12: SK22D: phages) with various K-12 strains with or without SK22D. For all tested K-12 strains a 100 to 10,000-fold increase of pfus/ml was observed when being incubated with phages (Figures 6A,B). Quite the contrary was detected when SK22D was added for K-12 protection. In the lambda-phage experimental set up, SK22D reduced the phage increase by K-12 strains up to 1,000 times (Figure 6A). When *stx*-phages were used, the protective effects of SK22D were even more pronounced. For the K-12 strain MG1655 the phage titer was 10,000 times lower compared to being incubated with phages alone and for HB101 and DH5α no phages were detected in the supernatant (Figure 6B). Next to the phage titer evaluation also the determination of the Stx level showed a dramatic increase in case of K-12 strains incubated with the EDL933 filtrate, which equally comprised *stx*-phages and the Stx protein. Likewise, to the phage results SK22D strongly interfered with this Stx accumulation after 24 h but showed no influence on the existing toxin level when being incubated with the phage filtrate alone for the same time period (Figure 6C). To investigate if other commensal *E. coli* have a similar impact on the K-12 protection, SE15 and IAI1 were co- (phages: commensal *E. coli*) and tri-cultured (phages: commensal *E. coli*: MG1655) with isolated phages. In case of the co-cultures with lambda-phages the commensals reduced the pfu by 10-fold (SE15) and 83-fold (IAI1) respectively. These strains could also interfere with the lambda-phage infection of MG1655 in tri- culture but not as efficient as EcN strain SK22D. This was resembled by a lambda-phage reduction of ~66-fold (SE15) and ~170-fold (IAI1) in the supernatant after incubation, whereas the reduction was ~ 640-fold when SK22D was present in the tri-culture (Figure 6D). In case of the co- and tri-cultures with *stx*-phages, the commensals could neither inactivate the phages in the co-culture nor could they interfere with the MG1655 mediated *stx*-phage increase (Figure 6E).

**Figure 7:**
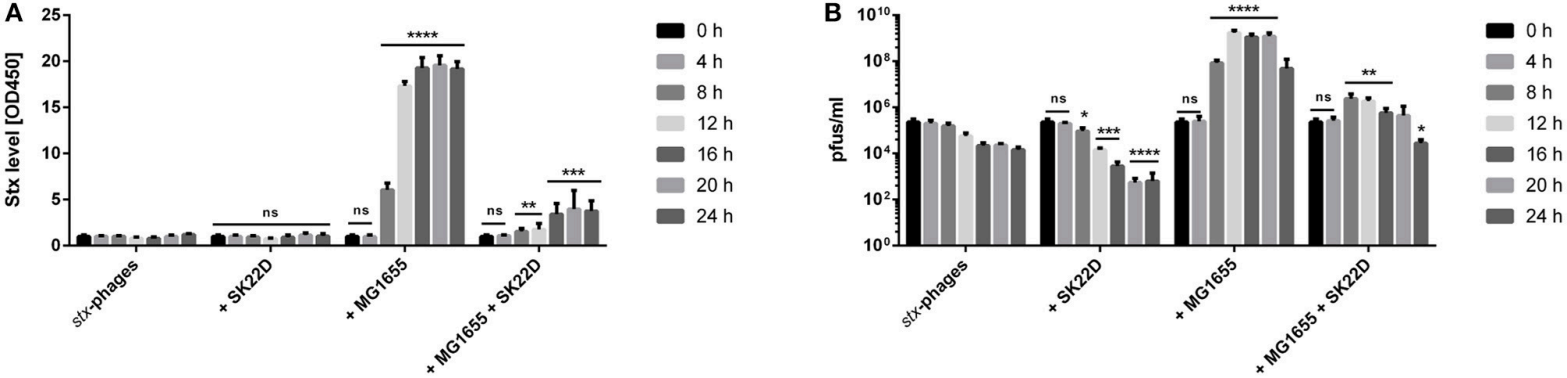
Kinetics of MG1655 *stx*-phage infection or protection by SK22D. *stx*-phages were incubated in mono-/co-/or tricultures with the microcin negative EcN strain SK22D and/or the K-12 strain MG1655 at a starting MOI of 1:10:10 (*stx*-phages: SK22D: MG1655) for 24 h, static. **(A)** The Stx level of the filtrate was determined with the Ridascreen® Verotoxin Elisa and **(B)** the pfus/ml were evaluated with a Phage Plaque Assay (PPA) every 4 h. ns, not significant, ^*^p < 0.05, ^**^p < 0.01, ^***^p < 0.001, and ^****^p < 0.0001.

Furthermore, the kinetics of the *stx*-phage titer as well as the Stx level in co- and tri-cultures were determined. The results depicted a Stx increase in the co-culture of isolated *stx*-phages and MG1655 after 8 h of incubation of ~600% and increased by ~2,500% after 16 h of incubation before it stagnated (Figure 7A). Similar kinetics were observed for the *stx*-phages cocultured with MG1655 with an increase of 530-fold after 8 h incubation, a peak after 12 h of incubation with an increase of 30,000-fold and a reduced *stx*-phage increase of 3,300-fold at the final time point after 24 h (Figure 7B). The presence of SK22D in the tri-culture (phages: SK22D: MG1655) on the other hand could strongly adverse these results with a Stx reduction of 75% and a *stx*-phage decrease of 35-fold after 8 h of incubation, a Stx decrease of 90%, and a 1,000-fold *stx*-phage reduction at the 12 h time point and a final toxin reduction of 80% and an ~2,000-fold decreased *stx*-phage titer at the 24 h time point compared to the co-culture with MG1655.

**Figure 8:**
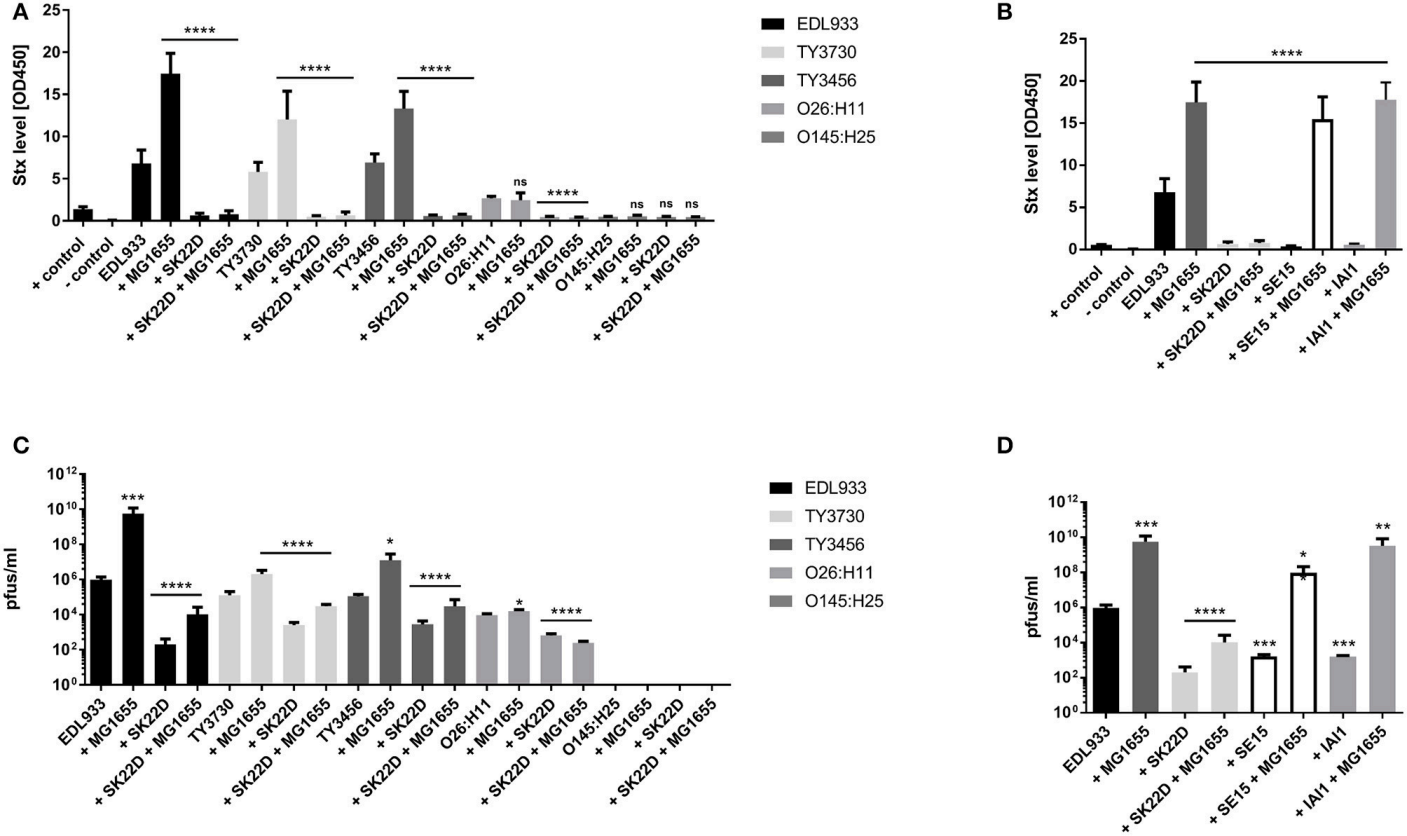
Protection of MG1655 Stx2 STEC phage infection. The Stx2 STEC strains EDL933, the two isolates from 2011 TY3730 and TY3456, O26:H11 and O145:H25 were incubated in mono-/co-/ and tri-cultures with SK22D and/or MG1655 in a ratio of 1:10:10. Additionally, EDL933 was incubated in mono-/co-/ and tri-culture with the commensal strains SE15 and IAI1 and/or MG1655 (1:10:10). After a 24 h, static incubation period the Stx level **(A,B)** and the phage titer **(C,D)** were detected in the sterile filtrate. The Stx level of the filtrate was determined with the Ridascreen® Verotoxin Elisa. LB Medium was used as negative control. The phage titer was determined with the Phage Plaque Assay (PPA). ns, not significant, ^*^p < 0.05, ^**^p < 0.01, ^***^p < 0.001, and ^****^p < 0.0001.

In a next, more fundamental approach, the protection of K-12 was tested with various Stx2 STEC strains, including two isolates of the outbreak from 2011 (Bielaszewska et al., 2011). In the coculture studies of the STEC strains EDL933 or the two O104:H4 strains (TY3730 and TY3456) with MG1655 (STEC: MG1655 = 1:10), the phage titer increased 10–1,000 times and the Stx level went up by about 100% (Figures 8A,C). In contrast, in the presence of SK22D the toxin level was reduced by about 90% regardless if the K-12 strain was present or not (STEC: MG1655: SK22D = 1:10:10). Furthermore, the phage titer was significantly decreased in the presence of the probiotic *E. coli*. For the STEC strain O26:H11 no Stx or phage rise was observed when being incubated with MG1655, but an 85% reduction of the toxin and a 40-fold decreased phage level was evidenced in the presence of SK22D. The *stx2* harboring strain O145:H25 was unable to produce phages which resulted in an absence of phage or toxin increase in the presence of MG1655. SE15 and IAI1 on the other hand, could interfere with the toxin production of the Stx2 STEC strain EDL933 in co-culture like SK22D (~90% reduced) but were incapable of interfering with the strong toxin increase of ~300% in tri-culture with MG1655 (Figure 8B). Additionally, the *stx*-phage level determination confirmed these results with a small *stx*-phage decrease of ~60-fold in the co-cultures but a ~120-/~3,000-fold (SE15/IAI1) increase of the *stx*-phages in the tri-culture compared to the EDL933 monoculture (Figure 8D).

**Figure 9:**
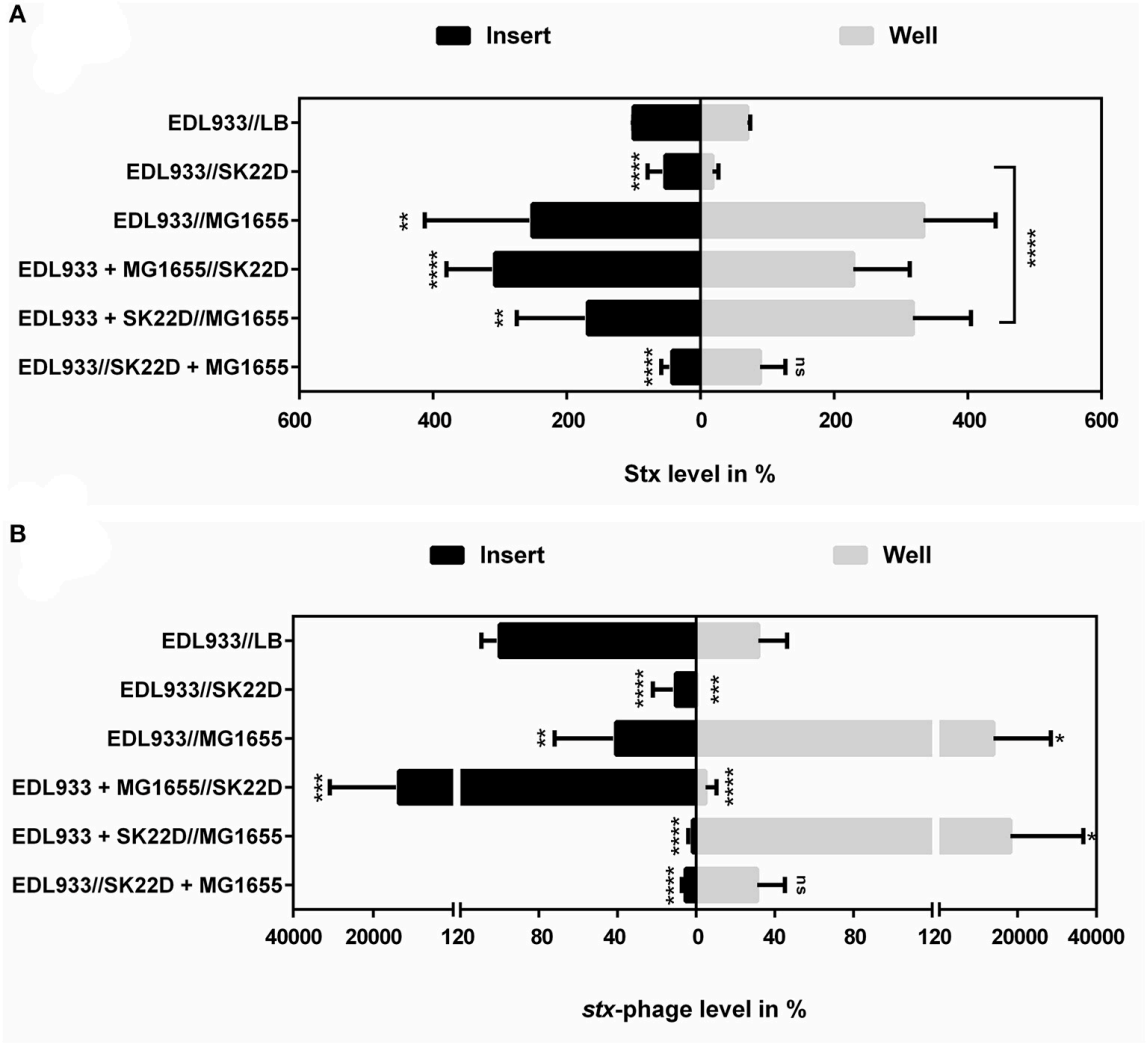
Protection of MG1655 in the Transwell system. The STEC strain EDL933, the microcin negative EcN mutant SK22D and the K-12 strain MG1655 were separated by a transwell membrane with a pore size of 0.4 μm in different combinations (1:10:10). After a 24 h, static incubation period the Stx level **(A)** and the phage titer **(B)** were detected in the sterile filtrate. The Stx level of the filtrate was determined with the Ridascreen® Verotoxin Elisa. LB Medium was used as negative control. The phage titer was determined with the Phage Plaque Assay (PPA). The values were converted to percentage compared to the respective compartment control. ns, not significant, ^*^p < 0.05, ^**^p < 0.01, ^***^p < 0.001, and ^****^p < 0.0001.

To resolve the K-12 protection mechanism by SK22D transwell experiments were performed in which *E. coli* strains were separated in different combinations by a membrane with a pore size of 0.4 μm (EDL933: MG1655: SK22D = 1:10:10). Stx level and phage determination from the well and the insert revealed that SK22D reduced the toxin production of EDL933 by 50% and the phage level by 90% without direct cell contact (Figure 9). In addition, it has been shown that in the presence of MG1655 the toxin and phage titer increased dramatically in all tested variations except if SK22D and MG1655 were in the same division.

Summarizing, the above listed experiments proved that SK22D can interfere with K-12 phage infection and associated therewith, the increase in phage number and toxin level.

## Discussion

The STEC strain O157:H7 is associated with the development of HUS and bloody diarrhea. However, also non-O157 STEC infections have been gaining importance as the cause of serious illness in the last two decades (Balamurugan et al., 2017). This rise of new STEC strains can be attributed to the fact that *stx* is encoded on a prophage (O'brien et al., 1984). The prophage evolves to a *stx* carrying phage during bacterial stress conditions and is subsequently released by bacterial lysis (Neely and Friedman, 1998; Wagner et al., 2002). These toxin harboring phages can thereafter integrate into the genome of another *E. coli* strain and transform it thereby to a new pathogenic lysogen (Schmidt, 2001). The treatment of STEC infected patients with antibiotics is a very controversial topic as such a treatment increases the stress conditions and release of the toxin (Pedersen et al., 2008). Therefore, alternative or supplementary strategies are needed. The probiotic strain EcN has been proven to interfere with the Stx release during *in vitro* studies (Rund et al., 2013; Mohsin et al., 2015). To take another important step toward the possibility of a supportive probiotic treatment of patients suffering from a STEC infection, it has by far the most importance to ensure that there is no possibility of EcN lysis or lysogeny by *stx*-phages.

EcN as a probiotic, has already been acclaimed for its fitness factors and for outcompeting other bacteria in the gut (Sonnenborn and Schulze, 2009; Deriu et al., 2013; Weiss, 2013). Previous studies performed in our lab illustrated the genome stability of EcN, as it was proven to be a weak recipient of foreign DNA by conjugation (data not shown). In this context, phage resistance will be an added benefit to the genome stability of EcN. The present study clearly demonstrated that EcN was never infected by the tested lambdoid phages (Figure 1). Phage-host coexistence in a complex environment like the gut is a widely accepted phenomenon contributing for interminable evolutions (Samson et al., 2013). Bacteria have been shown to evolve to counteract phage infection by adopting several strategies (Labrie et al., 2010). There are reported evidences of bacterial surface modifications like mutations in phage receptors or masking of these receptors by an exopolysaccharide layer or capsule, which would block the binding of phages. Bacteria were also discovered to block the entry of phage DNA by a superinfection exclusion system (Hofer et al., 1995; Bondy-Denomy et al., 2016) or to degrade the incoming phage DNA by an active restriction modification or the CRISPR-Cas system (Kim et al., 2012; Dupuis et al., 2013). One could also suspect several unidentified and undocumented defense mechanisms of bacteria, as it is an endless state of evolution. However, in case of EcN, results from this study strongly suggest that EcN uses more than one strategy against phage infections.

One of these mechanisms which is visible from the transcriptomic analysis is a prophage mediated defense against phage infection, which according to literature could be termed as superinfection immunity (Ranade and Poteete, 1993; Cumby et al., 2012). Specifically, the most upregulated gene was the early gene of the prophage 3 in EcN: *pr* (Figure 2B). This gene was chosen to be the candidate gene as it was previously reported to confer selective advantage to the lysogens by providing immunity against lytic infection (Ladero et al., 1998; Brussow et al., 2004). Consistent with these findings, we observed in our experiments that when *pr* was cloned into the phage sensitive MG1655 strain, lambda and *stx-*phages plated less efficiently on such a bacterial lawn (Figure 3). This effect could be explained as follows: as reported by Serra-Moreno and Muniesa (2008), the phage repressor could reduce the lysis of the cells and promote the lysogeny in these recombinant MG1655. However, in EcN no lysogens could been detected (Figures 1A,B). So along with the phage repressor, there must be some other factor in EcN that prevents the entry of the incoming phage DNA. The commensal control strains SE15 and IAI1 were also not infected by the *stx*- or lambda-phages, which was represented by the absence of a phage increase in the co-cultures with isolated phages (Figures 6D,E). Both strains were determined to carry lambdoid prophages in their genome comparable to the one of EcN, in contrast to MG1655 which encodes only a truncated lambdoid prophage (Table S2). Therefore, we hypothesize that the unidentified factor in EcN which is responsible for the complete protection against lambdoid phage infections could be the involvement of entire prophage 3 which remains to be tested. Owing to the finding, that one of EcN's prophages plays a key role in its resistance to phage infections, it was also imperative for us to investigate the activity of this prophage. As mentioned in the results section, prophage 3 was figured out to be an inactive/defective prophage. Hence, it can be hypothesized that the requisite for maintenance of this prophage in EcN's genome should be due to its significant contribution to the phage resistance of EcN. Thus, it will be of utmost interest in the future to elucidate the participation of other genes of prophage 3 in the phage resistance of EcN.

When being released from their host, phages pose a risk of infecting other bacteria present. Research regarding the defense of bacteria against phages mostly addresses the protection mechanisms towards phage infection as mentioned above. However, new scientific findings demonstrate that there are also other levels of bacterial defense against phages. Vidakovic et al. (2018) could show that amyloid fibers, which are part of the bacterial extracellular matrix, can bind phages and therefore, protect *E. coli* from phage infection in a cell distanced mode. In our experiments, it was shown that EcN could reduce the phage titer of both the lambdoid phages during static incubation.

It is known that lambda phages use the LamB receptor for binding to their host and releasing their DNA intracellularly for genomic integration (Chatterjee and Rothenberg, 2012). Sequence analysis of the phage susceptible K-12 strain MG1655 and EcN (Figure S2) demonstrate a 97% sequence identity of LamB. However, phages were detected by PCR to bind to both, the phage susceptible K-12 strain MG1655 and EcN (Figure 4C). These results suggest that the sequence differences in EcN LamB are not involved in protection from phage infection. In addition, we found that the expression of LamB from EcN or LamB from a K-12 strain in LamB negative K-12 strain did not result in any difference of phage adherence and infection (data not shown). The reduction of the lambda-phage titer by EcN could be traced back to the binding of the phages to the LamB of EcN which resulted in a reduction of free lambda-phages in the supernatant. This binding, confirmed by PCR (Figure 4C), was intensified in its prevalence by LamB upregulation with 0.2% maltose (Schwartz, 1976; Hoyland-Kroghsbo et al., 2013), and lost when the receptor was denaturated by heat treatment (Matsuura et al., 2015).

The *stx*-phage reduction by EcN exhibited different characteristics than the one of the lambda-phages. *stx*-phages use the YaeT receptor of *E. coli* as surface binding receptor (Smith et al., 2007). Anyhow, YaeT seemed to be uninvolved in the phage inactivation as EcN and MG1655 share a 100% YaeT sequence identity (Figure S2) and MG1655 was not able to reduce the phage titer when being heat killed due to probable receptor degradation, unlike the heat killed EcN. Moreover, the *stx*-phage inactivation properties of EcN were identified to be not common to all *E. coli* as the commensal strains SE15 and IAI1, which were also resistant toward a *stx*-phage infection, were incapable of reducing the pfus in the supernatant of the coculture after a 24 h incubation period (Figure 5D). As the phage reducing effect was lost after a Proteinase K treatment of the EcN heat-treated pellet, we conclude that the *stx*-phage reduction by EcN was achieved by a thermostable protein of EcN which is produced, or its production is increased after 12 h of static EcN incubation, the stationary *E. coli* growing phase. Therefore, genes upregulated in the stationary growth phase could be involved in *stx*-phage reduction like the biofilm which is formed during later growth phase (Grillo-Puertas et al., 2012). Different surface and extracellular matrix mutants of EcN tested were all able to lower the *stx*-phage titer like the EcN wt (data not shown), for which reason a combination of different surface or biofilm components cannot be excluded. Further studies are necessary to determine the exact mechanism of the *stx*-phage reduction by EcN.

STEC infected patients can show broad variations in disease progression (Paton and Paton, 1998). One assumption for the different outcomes is based on variations of their microbiome composition. A patient developing a HUS might have a microbiome with *stx*-phage susceptible *E. coli* whereas, the microbiome of a patient with less severe symptoms is composed of *stx*-phage resistant *E. coli*. This presumption is based on *in vivo* experiments in mice, where it was shown that phage susceptible strains in the intestine can lead to an increase in toxin accumulation (Gamage et al., 2003, 2006). Our *in vitro* studies, reconfirmed the lambdoid phage infection and connected to this an increase in the phage number by susceptible *E. coli* K-12 strains. In case of the *stx*-phages, the kinetics of infection depicted that MG1655 was turned into a *stx*-prophage lysogen before 8 h of incubation whereupon it became a Stx producer itself which was depicted by a strong toxin and phage increase during the co-incubation of MG1655 with *stx*-phages. More importantly, it was observed that the microcin negative EcN mutant SK22D (Patzer et al., 2003) could interfere with the phage infection of all K-12 strains and associated to this with the increase of the phage titer and the amplification of Stx. The interference with infection was shown to be contact dependent as SK22D could protect MG1655 from the infection only when being in the same transwell compartment. The commensal control strains SE15 and IAI1 were incapable of protecting the K-12 strain from *stx*-phage infection and underline thereby an EcN specific attribute. The protection of MG1655 was determined to be not based on a killing of the K-12 strain as the CFUs of MG1655 were comparable in the tri-culture with SK22D and the commensal controls (Figure S3). Possible modes of interference with the K-12 infection could be an integration of MG1655 in the biofilm network of EcN which has a pronounced effect in outcompeting pathogenic *E. coli* (Hancock et al., 2010). EcN's biofilm is different in its composition from the commensal control strains SE15 and IAI1 (e.g., discrepancies in the capsule, the LPS and flagella) and might as well have an effect on the lambdoid phages themselves. In case of the lambda-phages an absorbing of the free phages in the suspension due to EcN LamB receptor binding is another possible strategy for the detected K-12 protection.

The STEC strain isolated from the epidemic in 2011 (serotype O104:H4) led to a worsened clinical outcome for the patients (Frank et al., 2011). We wondered if EcN might protect *E. coli* K-12 strains against *stx*-phages form the O104:H4 strain as well as against *stx*-phages from other STEC strains. Still, co- and tri- culture studies with different Stx2 producing STEC strains unraveled the homogenous protection effect by SK22D and therewith a prominent reduction in phage and toxin increase. The protective effect seemed not to depend on the Stx2 STEC strain and was also achieved by the commensal control strains which suggests a similar interference with the Stx and *stx*-phage production as EcN, which is likely as both commensals were isolated from healthy individuals and are probably involved in the maintenance of the healthy microbiota (Touchon et al., 2009; Toh et al., 2010).

EcN's antagonistic activity against different pathogenic strains has been widely reported (Reissbrodt et al., 2009; Mohsin et al., 2015; Sonnenborn, 2016). In this study, we showed for the first-time phage resistance as a key probiotic and safety attribute of EcN. Apart from being protected against lambdoid phage infections, it appears from our results that EcN can also drastically reduce the phage numbers during co-incubation. Furthermore, EcN demonstrated interference with the infection of phage susceptible *E. coli* strains. This capacity could help in weakening the severity and progression of disease for a patient. For the reasons mentioned, we strongly promote further *in vivo* studies of EcN and STEC strains to investigate if EcN could be used in the prevention and disease treatment of a STEC infection in humans.

## Author contributions

Conceived and designed the experiments: TO, RvB. Performed the experiments: SB, MS. Analyzed the data: SB, MS, TO. Manuscript preparation: SB, MS, RB, KF, RvB, TO. Manuscript revision: RB, RvB, KF, TO. All authors read and approved the final manuscript.

### Conflict of interest statement

The study was supported by stipends to SB and MS by the companies Pharma-Zentrale GmbH and Ardeypharm GmbH. The remaining authors declare that the research was conducted in the absence of any commercial or financial relationships that could be construed as a potential conflict of interest.

## Footnotes

^1^Andrews S. FastQC: a quality control tool for high throughput sequence data. 2010. Available online at: http://www.bioinformatics.babraham.ac.uk/projects/fastqc

## Supplementary material

Figure S1 Gene regulations in the predicted prophages of EcN in presence of *stx-*phage.

Figure S2 Alignment of the amino acid sequence of the lambda phage (LamB) and *stx-*phage (YeaT) receptors of EcN with MG1655.

Figure S3 Growth determination of MG1655. MG1655 was cultured alone in LB Medium (MG1655) or with **(A)** lambda-phages (1:10) and **(B)** *stx*-phages (1:100) with or without SK22D or the commensal *E. coli* strains SE15 and IAI1 (MG1655: SK22D/commensals: 1:1). The CFUs of MG1655 were determined after a 24 h incubation period on ECC plates. ^***^*p* < 0.001, ^****^*p* < 0.0001.

Table S1 Table describing the prophages predicted in EcN by PHAST. Prophage 3 that is shown in this study to contribute for the superinfection immunity of EcN against lambdoid prophages.

Table S2 Presence of EcN's lambdoid prophage genes in the commensal strains SE15 and IAI1 and the K-12 strain MG1655. −, gene not present; +, gene present outside of a lambdoid prophage; P, gene encoded on a lambdoid prophage; ^*^Sequence not identical to EcN's lambdoid prophage.
