# Supplementary figures and images for: The Probiotic Escherichia coli Strain Nissle 1917 Combats Lambdoid Bacteriophages stx and λ
Source: Front Microbiol. 2018 May 29;9:929. doi: 10.3389/fmicb.2018.00929 (PMC5987069; doi:10.3389/fmicb.2018.00929)

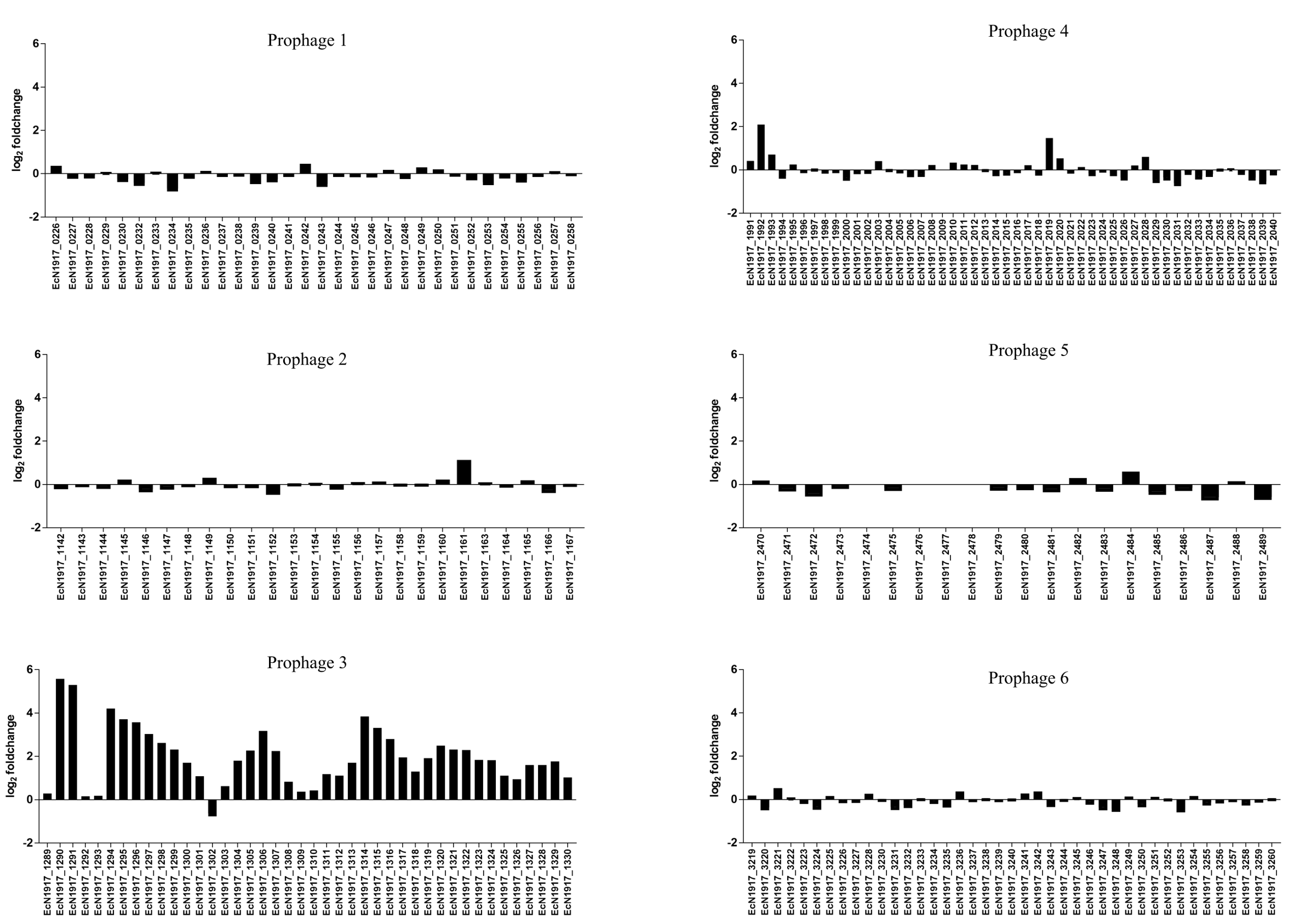

Supplement: Figure S1 — Gene regulations in the predicted prophages of EcN in presence of stx-phage. [file Image_1.tif]

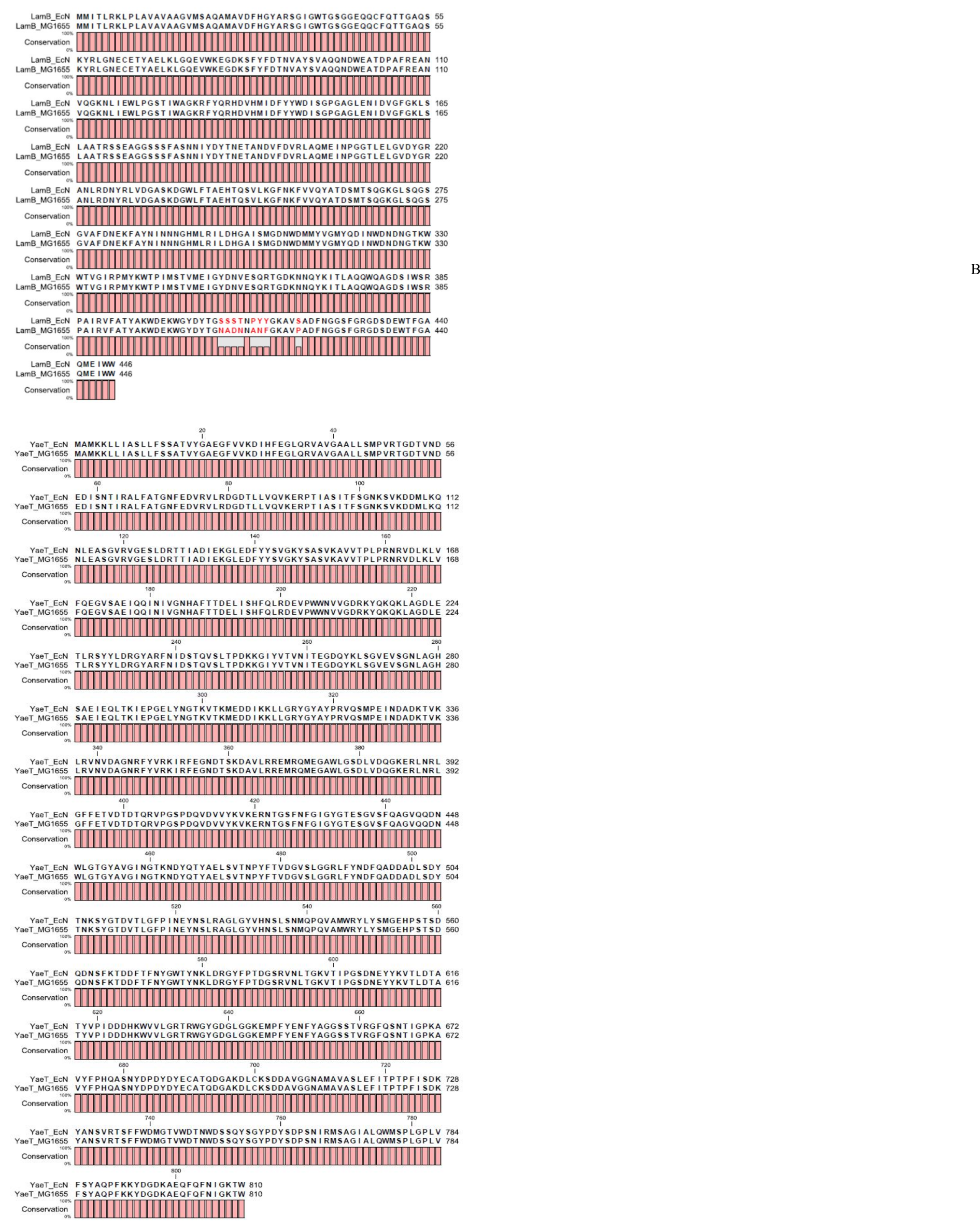

Supplement: Figure S2 — Alignment of the amino acid sequence of the lambda phage (LamB) and stx-phage (YeaT) receptors of EcN with MG1655. [file Image_2.tif]

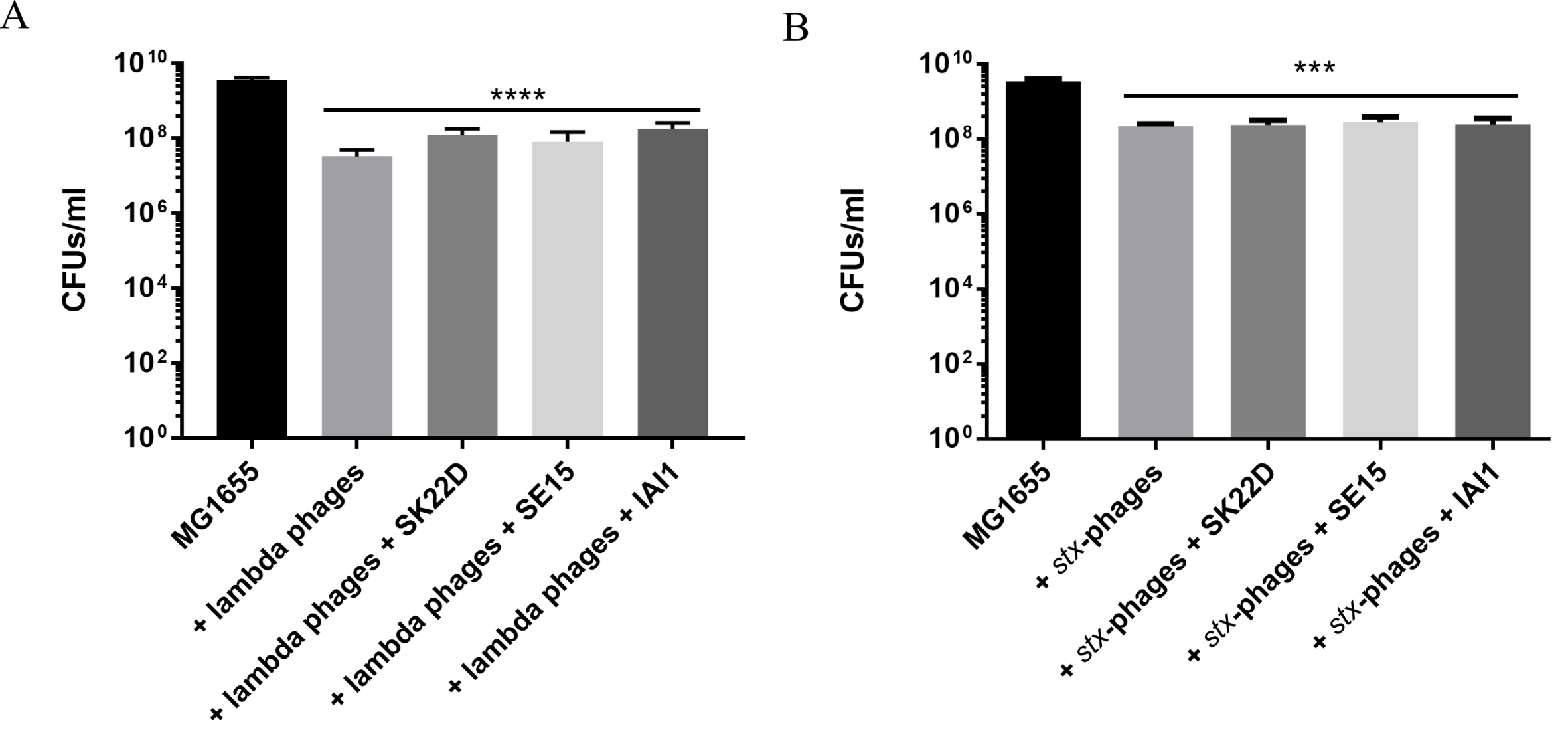

Supplement: Figure S3 — Growth determination of MG1655. MG1655 was cultured alone in LB Medium (MG1655) or with (A) lambda-phages (1:10) and (B) stx-phages (1:100) with or without SK22D or the commensal E. coli strains SE15 and IAI1 (MG1655: SK22D/commensals: 1:1). The CFUs of MG1655 were determined after a 24 h incubation period on ECC plates. ***p < 0.001, ****p < 0.0001. [file Image_3.tiff]
