# Supplementary material for: The Probiotic Escherichia coli Strain Nissle 1917 Combats Lambdoid Bacteriophages stx and λ
Source: Front Microbiol. 2018 May 29;9:929. doi: 10.3389/fmicb.2018.00929 (PMC5987069; doi:10.3389/fmicb.2018.00929)
Supplement: Table S1 — Table describing the prophages predicted in EcN by PHAST. Prophage 3 that is shown in this study to contribute for the superinfection immunity of EcN against lambdoid prophages. [file Table_1.docx]

|  | **REGION LENGTH** | **COMPLETENESS** | **SCORE** | **CDS/REGION POSITION/Gene ID** | **POSSIBLE PHAGE** | **GC %** |
| --- | --- | --- | --- | --- | --- | --- |
| **1** | 18.8Kb | questionable | 90 | 32/ 238183-257023  **0226 to 0258** | PHAGE_Klebsi_vB_KpnP_KpV475_NC_031025 | 50.41% |
| **2** | 15.1Kb | questionable | 90 | 25/ 1190179-1205354  **1142 to 1167** | PHAGE_Bacill_PfEFR_5_NC_031055 | 51.36% |
| **3** | 39.8Kb | intact | 150 | 49/ 1309318-1349204  **1284 to 1332** | PHAGE_Entero_lambda_NC_001416 | 50.93% |
| **4** | 59Kb | intact | 150 | 56/ 1995584-2054639  **1974 to 2044** | PHAGE_Entero_c_1_NC_019706 | 51.33% |
| **5** | 11.2Kb | incomplete | 40 | 15/ 2536491-2547748  **2470 to 2489** | PHAGE_Entero_N15_NC_001901 | 49.69% |
| **6** | 38.8Kb | intact | 100 | 21/ 3318669-3357505  **3219 to 3260** | PHAGE_Entero_P1_NC_005856 | 48.67% |
