# Supplementary material for: The Probiotic Escherichia coli Strain Nissle 1917 Combats Lambdoid Bacteriophages stx and λ
Source: Front Microbiol. 2018 May 29;9:929. doi: 10.3389/fmicb.2018.00929 (PMC5987069; doi:10.3389/fmicb.2018.00929)
Supplement: Table S2 — Presence of EcN's lambdoid prophage genes in the commensal strains SE15 and IAI1 and the K-12 strain MG1655. −, gene not present; +, gene present outside of a lambdoid prophage; P, gene encoded on a lambdoid prophage; *Sequence not identical to EcN's lambdoid prophage. [file Table_2.docx]

| **EcN** | **Description** | **SE15** | **IAI1** | **MG1655** |
| --- | --- | --- | --- | --- |
| **1289** | **Mobile element protein** | - | - | + |
| **1290** | **putative superinfection exclusion protein** | - | - | - |
| **1291** | **hypothetical protein** | - | - | - |
| **1292** | **hypothetical protein** | - | - | - |
| **1293** | **cI repressor protein** | P* | P* | - |
| **1294** | **Phage repressor** | P* | P* | - |
| **1295** | **Origin specific replication initiation factor** | P | P | - |
| **1296** | **Replication protein P** | P | P | - |
| **1297** | **Phage NinB DNA recombination** | P | P | - |
| **1298** | **Phage DNA N-6-adenine methyltransferase** | - | - | - |
| **1299** | **Phage NinX** | P | P | P |
| **1300** | **Crossover junction endodeoxyribonuclease rusA** | P | P | P |
| **1301** | **hypothetical protein** | P | P | P |
| **1302** | **Phage antitermination protein Q** | P | P | P |
| **1303** | **Outer membrane porin protein NmpC** | P | P | P |
| **1304** | **Phage holin** | P | P | P |
| **1305** | **Phage tail fiber protein** | P | P | P |
| **1306** | **Phage outer membrane lytic protein Rz3B Endopeptidase** | P | P | P |
| **1307** | **Lipoprotein Bor** | P | P | P |
| **1308** | **hypothetical protein** | - | - | - |
| **1309** | **hypothetical protein** | P | - | - |
| **1310** | **Terminase small subunit** | P | - | - |
| **1311** | **Phage terminase 2C large subunit** | P | - | - |
| **1312** | **Phage head-to-tail joining protein** | P | - | - |
| **1313** | **Phage portal protein** | P | - | - |
| **1314** | **Phage capsid and scaffold** | P | - | - |
| **1315** | **Head decoration protein** | P | - | - |
| **1316** | **Phage major capsid protein** | P | - | - |
| **1317** | **Phage DNA-packaging protein** | P | - | - |
| **1318** | **Phage capsid and scaffold** | P | P | - |
| **1319** | **Phage tail completion protein** | P | P | - |
| **1320** | **Phage minor tail protein** | P | P | - |
| **1321** | **Phage tail assembly** | P | P | - |
| **1322** | **Phage minor tail protein** | P | P | - |
| **1323** | **Phage minor tail protein** | P | P | - |
| **1324** | **Phage tail length tape-measure protein 1** | P | P | - |
| **1325** | **Phage minor tail protein** | P | P | - |
| **1326** | **Phage minor tail protein** | P | P | - |
| **1327** | **Phage tail assembly protein** | P | P | - |
| **1328** | **Phage tail assembly protein I** | P | P | - |
| **1329** | **Phage tail fiber protein** | P | P | - |
| **1330** | **Phage tail fiber protein** | P | P | - |
